# Supplementary material for: An adaptive extended radial basis function based interval analysis method for structural engineering solutions
Source: PLoS One. 2026 Apr 16;21(4):e0345852. doi: 10.1371/journal.pone.0345852 (PMC13086358; doi:10.1371/journal.pone.0345852)
Supplement: Attachment — Submitted filename: Response to Reviewer new.docx [file pone.0345852.s002.docx]

**Reviewer 4**

| Comment | Response |
| --- | --- |
| First, the manuscript introduced the adaptive extended radial basis function (E-RBF) to solve the uncertain interval structural problems. This application may have utility in the interval analysis method, but this work does not contribute significantly to the existing body of knowledge. | Thank you for your suggestion. The interval analysis method in this paper has significant contribution for the existing body of knowledge. In **Page 3, Line 56 to 67**, there is the introduction about the significance of interval analysis (Specifically, the interval method or interval analysis may be…). And in **Page 6, Line 117 to 125**, the advantage and contribution of this paper’s interval analysis has been explained. |
| The writing logic and readability of the manuscript should be further enhanced. The literature reviewed in the Introduction should be closely related to the topic and correctly summarized. The methodology should focus on original algorithms and ideas to highlight the authors’ contribution. | Thank you for your suggestion. We have rearrange the introduction part to ensure a clear logical main line. The revised part is shown form **Page 3 to 6** (However, this accurate function necessitates a great amount of statistical information for experimental data or simulation data…). |
| In Numerical problems, a practical engineering scenario should be provided to demonstrate the effectiveness of the proposed method. In addition, the comparison methods chosen between different problems are inconsistent and lack a detailed, quantitative description of the results. | Thank you for your suggestion, and we have made all the comparison results in the same type (See **Table 8 to 11**). And we add a practical engineering composite shell example in **Page 31 to 34, Line 518 to 556**. |
| There are garbled codes in some formulas. For example, Eq. (1), (2), and (4). | Thank you for your suggestion, and we have revised the Equation (See **Equation (1), (2), (4), (28), (30), (31), (32)**). |
