# Supplementary material for: An adaptive extended radial basis function based interval analysis method for structural engineering solutions
Source: PLoS One. 2026 Apr 16;21(4):e0345852. doi: 10.1371/journal.pone.0345852 (PMC13086358; doi:10.1371/journal.pone.0345852)
Supplement: Attachment — Submitted filename: Response to Reviewers.docx [file pone.0345852.s003.docx]

**Reviewer 2**

| Comment | Response |
| --- | --- |
| I recommend expanding the abstract to within 200–250 words, focusing on the core aim, methodology, key results, and conclusions. | Thank you for your support. And I have revised the Abstract to 200-250 words. See Page 1 and 2, Line 16 to Line 32. |
| Revise in the abstract (To test the accuracy and efficiency of the E-RBF with adjusted widths, six numerical functions are chosen.). | Thank you for your suggestion, we revised this sentence in the abstract.  See Page 1 Line 24 to 26. |
| The manuscript contains numerous grammatical errors. | Thank you for your suggestion, we have comprehensively optimized the article writing and grammar issues. |
| The paper addresses a significant challenge in structural engineering: handling uncertainties in interval analysis for complex systems. The introduction effectively highlights the importance of uncertainty quantification, distinguishing between aleatory and epistemic uncertainties, and justifies the need for non-probabilistic methods like interval analysis. However, the motivation for focusing specifically on the adaptive E-RBF method could be strengthened by explicitly stating how it addresses limitations of existing methods (e.g., computational inefficiency or inaccuracy in large interval ranges) earlier in the introduction https://doi.org/10.1016/j.jobe.2025.113302 - https://doi.org/10.1038/s41598-025-06725-z | Thank you for your suggestion. We added an expression about motivation in the Introduction.  See Page 4 to 5, Line 86 to 93.  "However, double-loop or nested-loop optimization-based process means more intensive computation. In order to save the computational cost, the approximation or surrogate model should be introduced for the nested-loop or double-loop interval analysis. M. Abdellatief [53] presented a machine-learning technique to address the limitations of the complex, non-linear interactions between different mixing design parameters. To balance accuracy and efficiency, there is artificial intelligence method to predict the early-age compressive strength for optimal mix design [54]. And these reviews are the motivation of the E-RBF method." |
| The novelty is well-demonstrated through comparisons with traditional RBF and other methods, though the paper could further clarify how the E-RBF outperforms other surrogate models (e.g., Kriging or response surface methods) in specific contexts. | Thanks for the comment. This will be our next goal.  See Page 37 and 38, Line 628 to 630. |
| The objective, methodology, and results should be better described, discussed and justified. | Thanks for the comment. I have revised the objective, methodology, and results. |
| The manuscript contains excessive abbreviations. Only the necessary ones should be retained. | Thanks for the comment. I have deleted the excessive abbreviations. |
| Equations (1) to (32) provide a clear derivation of the E-RBF and its application to interval analysis. However, the explanation of the pseudo-inverse approach for solving underdetermined systems (Equation 19) is brief and lacks discussion on numerical stability or potential issues (e.g., ill-conditioning of the matrix A). | Thank you for your comment.  We add an expression in Page 18, Line 312 to 313. |
| The paper lacks quantitative data on actual computational time or resource usage, which would provide a more concrete evaluation of efficiency. Including such metrics would strengthen the claims. | Thank you for your comment.  We add the computational resources. See Table 9 in Page 27, Line 453 and Table 12 in Page 35, Line 581 |
| Revise Fig. 5. | Thank you for your comment.  We have revised Fig. 5 in Page 16, Line 280. |
| Ensure the accurate and consistent use of superscripts and subscripts throughout the manuscript. Any improper formatting or irregularities may indicate that the text was generated or modified using AI tools. | Thank you for your comment.  We have ensured all the superscripts and subscripts. |
| Compare the results to contemporary work in a separate section, namely, comparison to contemporary work. | Thank you for your comment.  We have separate this section, Page 26 to 35, Line 441 to 582. |
| For the conclusion it is so short; please update it and add academic numbers and percentages. | Thank you for your comment.  We have revised the conclusion, Page 35 to 38, Line 584 to 624. |
| Update all references to ensure they are current and relevant. | Thank you for your comment. We have updated the references. |

**Reviewer 3**

| Comment | Response |
| --- | --- |
| A thorough language edit is required. Consider using professional editing services before resubmission. | Thank you for your support. We have updated the thorough language. |
| Lack of Benchmark Comparisons. The method is mainly compared to Zhao’s work; broader comparisons to other interval methods, e.g., Kriging, fuzzy-set methods, would strengthen the paper. Please include additional benchmarks or at least a discussion on how the proposed method compares in broader contexts. | Thank you for your suggestion. we have added a comparison between accurate function and adaptive interval analysis method in RBF.  See Page 27 and 28 Line 451 to 465. |
| Ensure all figures and tables are included, clearly labelled, and cross-referenced correctly in the text. | Thank you for your suggestion, we have ensured all the figrures and tables. |
| The mathematics is extensive but lacks deeper theoretical validation (e.g., convergence proof, sensitivity analysis). Add theoretical justification or boundary conditions under which E-RBF guarantees convergence or uniqueness. | Thank you for your suggestion. We have added a comparison between RBF and E-RBF, See Page 19 to 20 and 27 to 28. |
